# (*R*_P_,*R*_P_)-Bis[(3-menthyloxy)(phenyl)­phosphino­yl] disulfide

**DOI:** 10.1107/S1600536811038165

**Published:** 2011-09-30

**Authors:** Zhong-Yuan Xu, Chang-Qiu Zhao

**Affiliations:** a College of Chemistry and Chemical Engineering, Liaocheng University, Shandong 252059, People’s Republic of China

## Abstract

The molecule of the title compound, C_32_H_48_O_4_P_2_S_2_, has 2 symmetry, the mid-point of the S—S bond being located on a twofold rotation axis. The two tetra­hedral P units are linked by a S—S bond with a P—S—S—P torsion angle is 131.19 (6)°. The dihedral angle between two phenyl rings is 12.66 (13)°. The cyclo­hexane ring of the menthoxyl group displays a chair conformation. Weak inter­molecular C—H⋯O hydrogen bonding is present in the crystal structure.

## Related literature

For general background to chiral phospho­rus compounds, see: Perlikowska & Daran (2004 ▶).
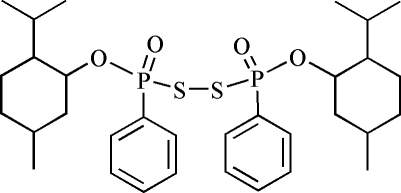


## Experimental

### 

#### Crystal data

C_32_H_48_O_4_P_2_S_2_
*M* *_r_* = 622.76
Orthorhombic, 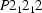

*a* = 9.9910 (9) Å
*b* = 18.9100 (17) Å
*c* = 8.9747 (7) Å
*V* = 1695.6 (3) Å^3^
*Z* = 2
Mo *K*α radiation
μ = 0.28 mm^−1^
*T* = 298 K
0.40 × 0.28 × 0.16 mm

#### Data collection

Bruker SMART 1000 CCD area-detector diffractometer
Absorption correction: multi-scan (*SADABS*; Sheldrick, 1996 ▶) *T*_min_ = 0.895, *T*_max_ = 0.956
7818 measured reflections
2989 independent reflections
2141 reflections with *I* > 2σ(*I*)
*R*_int_ = 0.043

#### Refinement

*R*[*F* ^2^ > 2σ(*F* ^2^)] = 0.041
*wR*(*F* ^2^) = 0.080
*S* = 0.91
2989 reflections
184 parameters
H-atom parameters constrained
Δρ_max_ = 0.33 e Å^−3^
Δρ_min_ = −0.16 e Å^−3^
Absolute structure: Flack (1983 ▶), 1736 Friedel pairs
Flack parameter: −0.10 (10)

### 

Data collection: *SMART* (Bruker, 2007 ▶); cell refinement: *SAINT* (Bruker, 2007 ▶); data reduction: *SAINT*; program(s) used to solve structure: *SHELXS97* (Sheldrick, 2008 ▶); program(s) used to refine structure: *SHELXL97* (Sheldrick, 2008 ▶); molecular graphics: *SHELXTL* (Sheldrick, 2008 ▶); software used to prepare material for publication: *SHELXTL*.

## Supplementary Material

Crystal structure: contains datablock(s) I, global. DOI: 10.1107/S1600536811038165/xu5323sup1.cif

Structure factors: contains datablock(s) I. DOI: 10.1107/S1600536811038165/xu5323Isup2.hkl

Supplementary material file. DOI: 10.1107/S1600536811038165/xu5323Isup3.cml

Additional supplementary materials:  crystallographic information; 3D view; checkCIF report

## Figures and Tables

**Table 1:** Hydrogen-bond geometry (Å, °)

*D*—H⋯*A*	*D*—H	H⋯*A*	*D*⋯*A*	*D*—H⋯*A*
C6—H6*B*⋯O2^i^	0.97	2.59	3.527 (3)	162

Symmetry code: (i) 
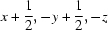
.

**Fig. 1.**
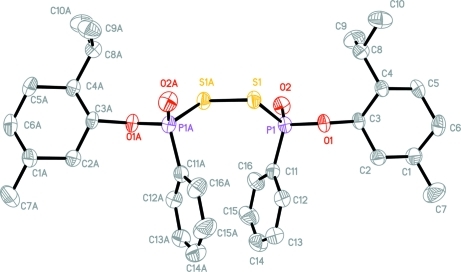
The molecular structure of the compound. H atoms have been omitted for clarity.

## supplementary crystallographic information

### Comment

Chiral phosphorus compounds have been widely used in both chemistry and biology (Perlikowska & Daran, 2004). The *P*-chiral title compound was synthesized by (*R*_p_)-*O*-menthyl phenylphosphonothioate and sulfuryl chloride. The fully extended substituents phenyl, menthoxy link to phosphorus atom, and the two phosphorus atoms are connected by dithio bond to form two similar *P*-centered irregular tetrahedrons. The angle of O2—P—S1 and O1—P—S1 are 113.31 (9) ° and 95.11 (7) °. Intermolecular C6—H6···O2 hydrogen bonds is observed in the crystal structure (Table 1).

### Experimental

Sulfuryl chloride (0.3 mmol) was added to a stirred ether solution of (*R*_p_)-*O*-menthyl phenylphosphonothioate (0.6 mmol) in a Schlenk tube under nitrogen, and the mixture was stirred for 4 h at 273 K. After washing with water and removing solvents, the resulting residue was purified by preparative TLC on silica gel to afford optically pure product. The crystal suit for X-ray diffraction was obtained from recrystallization with ethyl ether.

### Refinement

All H atoms attached to C atoms were fixed geometrically and treated as riding with C—H = 0.93 - 0.98 Å, with *U*_iso_(H) = 1.5 *U*_eq_(methyl) and *U*_iso_(H) = 1.2 *U*_eq_(C) for all other H atoms.

### Crystal data

**Table:**

C_32_H_48_O_4_P_2_S_2_	*F*(000) = 668
*M**_r_* = 622.76	*D*_x_ = 1.220 Mg m^−^^3^
Orthorhombic, *P*2_1_2_1_2	Mo *K*α radiation, λ = 0.71073 Å
Hall symbol: P 2 2ab	Cell parameters from 1878 reflections
*a* = 9.9910 (9) Å	θ = 3.1–22.1°
*b* = 18.9100 (17) Å	µ = 0.28 mm^−^^1^
*c* = 8.9747 (7) Å	*T* = 298 K
*V* = 1695.6 (3) Å^3^	Block, colorless
*Z* = 2	0.40 × 0.28 × 0.16 mm

### Data collection

**Table:**

Bruker SMART 1000 CCD area-detector diffractometer	2989 independent reflections
Radiation source: fine-focus sealed tube	2141 reflections with *I* > 2σ(*I*)
graphite	*R*_int_ = 0.043
φ and ω scans	θ_max_ = 25.0°, θ_min_ = 2.2°
Absorption correction: multi-scan (*SADABS*; Sheldrick, 1996)	*h* = −11→9
*T*_min_ = 0.895, *T*_max_ = 0.956	*k* = −15→22
7818 measured reflections	*l* = −10→10

### Refinement

**Table:**

Refinement on *F*^2^	Secondary atom site location: difference Fourier map
Least-squares matrix: full	Hydrogen site location: inferred from neighbouring sites
*R*[*F*^2^ > 2σ(*F*^2^)] = 0.041	H-atom parameters constrained
*wR*(*F*^2^) = 0.080	*w* = 1/[σ^2^(*F*_o_^2^) + (0.0327*P*)^2^] where *P* = (*F*_o_^2^ + 2*F*_c_^2^)/3
*S* = 0.91	(Δ/σ)_max_ = 0.001
2989 reflections	Δρ_max_ = 0.33 e Å^−^^3^
184 parameters	Δρ_min_ = −0.16 e Å^−^^3^
0 restraints	Absolute structure: Flack (1983), 1736 Friedel pairs
Primary atom site location: structure-invariant direct methods	Flack parameter: −0.10 (10)

### Special details

**Table:**

Geometry. All e.s.d.'s (except the e.s.d. in the dihedral angle between two l.s. planes) are estimated using the full covariance matrix. The cell e.s.d.'s are taken into account individually in the estimation of e.s.d.'s in distances, angles and torsion angles; correlations between e.s.d.'s in cell parameters are only used when they are defined by crystal symmetry. An approximate (isotropic) treatment of cell e.s.d.'s is used for estimating e.s.d.'s involving l.s. planes.
Refinement. Refinement of *F*^2^ against ALL reflections. The weighted *R*-factor *wR* and goodness of fit *S* are based on *F*^2^, conventional *R*-factors *R* are based on *F*, with *F* set to zero for negative *F*^2^. The threshold expression of *F*^2^ > σ(*F*^2^) is used only for calculating *R*-factors(gt) *etc*. and is not relevant to the choice of reflections for refinement. *R*-factors based on *F*^2^ are statistically about twice as large as those based on *F*, and *R*- factors based on ALL data will be even larger.

### Fractional atomic coordinates and isotropic or equivalent isotropic displacement parameters (Å^2^)

**Table:**

	*x*	*y*	*z*	*U*_iso_*/*U*_eq_	
P1	0.89547 (8)	0.39154 (4)	0.02808 (9)	0.0522 (2)	
S1	1.05009 (7)	0.45208 (4)	−0.06827 (9)	0.0569 (2)	
O1	0.97493 (16)	0.31924 (8)	0.0227 (2)	0.0530 (5)	
O2	0.76990 (17)	0.39533 (10)	−0.0544 (2)	0.0674 (6)	
C1	0.8604 (3)	0.13924 (16)	0.1576 (4)	0.0695 (10)	
H1	0.7641	0.1447	0.1399	0.083*	
C2	0.9227 (3)	0.21278 (15)	0.1605 (3)	0.0606 (9)	
H2A	0.8801	0.2408	0.2376	0.073*	
H2B	1.0171	0.2089	0.1847	0.073*	
C3	0.9073 (3)	0.24950 (13)	0.0128 (3)	0.0479 (7)	
H3	0.8118	0.2572	−0.0061	0.057*	
C4	0.9659 (3)	0.20881 (14)	−0.1165 (3)	0.0494 (7)	
H4	1.0612	0.2021	−0.0953	0.059*	
C5	0.9012 (3)	0.13521 (15)	−0.1183 (4)	0.0633 (9)	
H5A	0.8066	0.1399	−0.1405	0.076*	
H5B	0.9417	0.1071	−0.1966	0.076*	
C6	0.9178 (3)	0.09692 (14)	0.0308 (4)	0.0694 (9)	
H6A	0.8732	0.0514	0.0259	0.083*	
H6B	1.0122	0.0885	0.0490	0.083*	
C7	0.8786 (5)	0.10092 (19)	0.3060 (5)	0.1305 (18)	
H7A	0.8379	0.0550	0.3005	0.196*	
H7B	0.8371	0.1278	0.3841	0.196*	
H7C	0.9724	0.0958	0.3268	0.196*	
C8	0.9561 (3)	0.24793 (17)	−0.2669 (3)	0.0649 (9)	
H8	0.9846	0.2967	−0.2479	0.078*	
C9	0.8146 (3)	0.2525 (2)	−0.3295 (4)	0.0916 (12)	
H9A	0.8159	0.2785	−0.4214	0.137*	
H9B	0.7577	0.2762	−0.2592	0.137*	
H9C	0.7811	0.2057	−0.3473	0.137*	
C10	1.0536 (4)	0.2180 (2)	−0.3807 (4)	0.0975 (12)	
H10A	1.1426	0.2190	−0.3404	0.146*	
H10B	1.0505	0.2461	−0.4698	0.146*	
H10C	1.0293	0.1701	−0.4037	0.146*	
C11	0.8768 (3)	0.41490 (14)	0.2192 (3)	0.0553 (8)	
C12	0.9730 (3)	0.39822 (16)	0.3243 (4)	0.0703 (9)	
H12	1.0529	0.3774	0.2941	0.084*	
C13	0.9515 (5)	0.41225 (17)	0.4747 (4)	0.0896 (11)	
H13	1.0163	0.4012	0.5454	0.108*	
C14	0.8321 (5)	0.4428 (2)	0.5164 (5)	0.0997 (13)	
H14	0.8155	0.4510	0.6169	0.120*	
C15	0.7377 (4)	0.4614 (2)	0.4142 (5)	0.0967 (13)	
H15	0.6592	0.4836	0.4444	0.116*	
C16	0.7594 (3)	0.44701 (18)	0.2661 (4)	0.0765 (10)	
H16	0.6944	0.4590	0.1963	0.092*	

### Atomic displacement parameters (Å^2^)

**Table:**

	*U*^11^	*U*^22^	*U*^33^	*U*^12^	*U*^13^	*U*^23^
P1	0.0510 (4)	0.0433 (4)	0.0622 (6)	−0.0001 (3)	0.0007 (4)	−0.0039 (4)
S1	0.0600 (5)	0.0421 (4)	0.0685 (6)	0.0028 (4)	0.0114 (4)	−0.0015 (4)
O1	0.0520 (10)	0.0356 (10)	0.0714 (14)	−0.0035 (8)	−0.0013 (10)	−0.0056 (10)
O2	0.0521 (12)	0.0702 (13)	0.0800 (17)	0.0056 (10)	−0.0127 (11)	−0.0062 (14)
C1	0.081 (3)	0.057 (2)	0.070 (2)	−0.0237 (17)	−0.0024 (19)	0.002 (2)
C2	0.069 (2)	0.0560 (19)	0.057 (2)	−0.0117 (16)	−0.0014 (16)	−0.0027 (17)
C3	0.0434 (14)	0.0371 (14)	0.063 (2)	−0.0067 (12)	−0.0046 (15)	−0.0061 (15)
C4	0.0423 (16)	0.0451 (16)	0.061 (2)	0.0016 (14)	−0.0003 (15)	−0.0056 (15)
C5	0.065 (2)	0.0479 (19)	0.076 (2)	−0.0032 (16)	0.0040 (19)	−0.0192 (18)
C6	0.0625 (19)	0.0420 (17)	0.104 (3)	−0.0090 (15)	−0.011 (2)	0.003 (2)
C7	0.202 (5)	0.096 (3)	0.093 (3)	−0.056 (4)	−0.014 (3)	0.035 (3)
C8	0.076 (2)	0.060 (2)	0.059 (2)	−0.0048 (19)	−0.0015 (19)	−0.0016 (18)
C9	0.082 (3)	0.114 (3)	0.079 (3)	0.001 (2)	−0.016 (2)	0.018 (3)
C10	0.092 (3)	0.131 (3)	0.070 (2)	0.004 (2)	0.025 (2)	−0.004 (2)
C11	0.059 (2)	0.0455 (18)	0.061 (2)	−0.0014 (16)	0.0015 (17)	0.0014 (15)
C12	0.088 (2)	0.058 (2)	0.065 (2)	0.0027 (19)	0.001 (2)	−0.0030 (19)
C13	0.128 (3)	0.074 (2)	0.066 (3)	0.000 (2)	−0.009 (3)	0.003 (2)
C14	0.139 (4)	0.086 (3)	0.074 (3)	−0.011 (3)	0.031 (3)	−0.012 (3)
C15	0.096 (3)	0.104 (3)	0.090 (4)	0.009 (3)	0.030 (3)	−0.028 (3)
C16	0.072 (2)	0.075 (2)	0.082 (3)	0.009 (2)	0.0082 (19)	−0.017 (2)

### Geometric parameters (Å, °)

**Table:**

P1—O2	1.4583 (19)	C7—H7A	0.9600
P1—O1	1.5817 (17)	C7—H7B	0.9600
P1—C11	1.781 (3)	C7—H7C	0.9600
P1—S1	2.1083 (10)	C8—C10	1.520 (4)
S1—S1^i^	2.0703 (14)	C8—C9	1.524 (4)
O1—C3	1.484 (3)	C8—H8	0.9800
C1—C6	1.505 (4)	C9—H9A	0.9600
C1—C2	1.524 (4)	C9—H9B	0.9600
C1—C7	1.528 (4)	C9—H9C	0.9600
C1—H1	0.9800	C10—H10A	0.9600
C2—C3	1.505 (4)	C10—H10B	0.9600
C2—H2A	0.9700	C10—H10C	0.9600
C2—H2B	0.9700	C11—C12	1.383 (4)
C3—C4	1.510 (4)	C11—C16	1.387 (4)
C3—H3	0.9800	C12—C13	1.392 (5)
C4—C5	1.535 (4)	C12—H12	0.9300
C4—C8	1.542 (4)	C13—C14	1.378 (5)
C4—H4	0.9800	C13—H13	0.9300
C5—C6	1.530 (4)	C14—C15	1.361 (5)
C5—H5A	0.9700	C14—H14	0.9300
C5—H5B	0.9700	C15—C16	1.374 (5)
C6—H6A	0.9700	C15—H15	0.9300
C6—H6B	0.9700	C16—H16	0.9300
			
O2—P1—O1	117.31 (11)	H6A—C6—H6B	108.0
O2—P1—C11	112.74 (14)	C1—C7—H7A	109.5
O1—P1—C11	107.23 (13)	C1—C7—H7B	109.5
O2—P1—S1	113.30 (10)	H7A—C7—H7B	109.5
O1—P1—S1	95.12 (7)	C1—C7—H7C	109.5
C11—P1—S1	109.70 (10)	H7A—C7—H7C	109.5
S1^i^—S1—P1	96.96 (5)	H7B—C7—H7C	109.5
C3—O1—P1	122.78 (15)	C10—C8—C9	111.6 (3)
C6—C1—C2	110.0 (3)	C10—C8—C4	111.7 (3)
C6—C1—C7	111.2 (3)	C9—C8—C4	114.1 (3)
C2—C1—C7	111.6 (3)	C10—C8—H8	106.3
C6—C1—H1	107.9	C9—C8—H8	106.3
C2—C1—H1	107.9	C4—C8—H8	106.3
C7—C1—H1	107.9	C8—C9—H9A	109.5
C3—C2—C1	111.3 (2)	C8—C9—H9B	109.5
C3—C2—H2A	109.4	H9A—C9—H9B	109.5
C1—C2—H2A	109.4	C8—C9—H9C	109.5
C3—C2—H2B	109.4	H9A—C9—H9C	109.5
C1—C2—H2B	109.4	H9B—C9—H9C	109.5
H2A—C2—H2B	108.0	C8—C10—H10A	109.5
O1—C3—C2	108.1 (2)	C8—C10—H10B	109.5
O1—C3—C4	108.8 (2)	H10A—C10—H10B	109.5
C2—C3—C4	113.7 (2)	C8—C10—H10C	109.5
O1—C3—H3	108.7	H10A—C10—H10C	109.5
C2—C3—H3	108.7	H10B—C10—H10C	109.5
C4—C3—H3	108.7	C12—C11—C16	118.8 (3)
C3—C4—C5	107.9 (2)	C12—C11—P1	121.8 (2)
C3—C4—C8	113.8 (2)	C16—C11—P1	119.3 (3)
C5—C4—C8	113.5 (3)	C11—C12—C13	120.6 (3)
C3—C4—H4	107.1	C11—C12—H12	119.7
C5—C4—H4	107.1	C13—C12—H12	119.7
C8—C4—H4	107.1	C14—C13—C12	118.5 (4)
C6—C5—C4	112.0 (2)	C14—C13—H13	120.7
C6—C5—H5A	109.2	C12—C13—H13	120.7
C4—C5—H5A	109.2	C15—C14—C13	121.6 (4)
C6—C5—H5B	109.2	C15—C14—H14	119.2
C4—C5—H5B	109.2	C13—C14—H14	119.2
H5A—C5—H5B	107.9	C14—C15—C16	119.5 (4)
C1—C6—C5	111.6 (2)	C14—C15—H15	120.3
C1—C6—H6A	109.3	C16—C15—H15	120.3
C5—C6—H6A	109.3	C15—C16—C11	120.9 (4)
C1—C6—H6B	109.3	C15—C16—H16	119.6
C5—C6—H6B	109.3	C11—C16—H16	119.6
			
O2—P1—S1—S1^i^	56.22 (9)	C4—C5—C6—C1	57.2 (3)
O1—P1—S1—S1^i^	178.92 (8)	C3—C4—C8—C10	161.2 (3)
C11—P1—S1—S1^i^	−70.73 (11)	C5—C4—C8—C10	−74.9 (3)
O2—P1—O1—C3	−33.0 (3)	C3—C4—C8—C9	−71.2 (3)
C11—P1—O1—C3	95.0 (2)	C5—C4—C8—C9	52.8 (4)
S1—P1—O1—C3	−152.5 (2)	O2—P1—C11—C12	163.5 (2)
C6—C1—C2—C3	54.6 (4)	O1—P1—C11—C12	32.9 (3)
C7—C1—C2—C3	178.6 (3)	S1—P1—C11—C12	−69.2 (3)
P1—O1—C3—C2	−108.6 (2)	O2—P1—C11—C16	−12.7 (3)
P1—O1—C3—C4	127.4 (2)	O1—P1—C11—C16	−143.3 (2)
C1—C2—C3—O1	−177.8 (2)	S1—P1—C11—C16	114.6 (2)
C1—C2—C3—C4	−56.8 (3)	C16—C11—C12—C13	1.1 (5)
O1—C3—C4—C5	176.3 (2)	P1—C11—C12—C13	−175.1 (2)
C2—C3—C4—C5	55.8 (3)	C11—C12—C13—C14	0.2 (5)
O1—C3—C4—C8	−56.8 (3)	C12—C13—C14—C15	−2.1 (6)
C2—C3—C4—C8	−177.3 (2)	C13—C14—C15—C16	2.5 (7)
C3—C4—C5—C6	−55.2 (3)	C14—C15—C16—C11	−1.0 (6)
C8—C4—C5—C6	177.7 (3)	C12—C11—C16—C15	−0.8 (5)
C2—C1—C6—C5	−55.2 (3)	P1—C11—C16—C15	175.5 (3)
C7—C1—C6—C5	−179.5 (3)		

Symmetry codes: (i) −*x*+2, −*y*+1, *z*.

### Hydrogen-bond geometry (Å, °)

**Table:**

*D*—H···*A*	*D*—H	H···*A*	*D*···*A*	*D*—H···*A*
C6—H6B···O2^ii^	0.97	2.59	3.527 (3)	162.

Symmetry codes: (ii) *x*+1/2, −*y*+1/2, −*z*.
